# Pan-cancer EMT-signature identifies RBM47 down-regulation during colorectal cancer progression

**DOI:** 10.1038/s41598-017-04234-2

**Published:** 2017-07-05

**Authors:** Matjaz Rokavec, Markus Kaller, David Horst, Heiko Hermeking

**Affiliations:** 1 Experimental and Molecular Pathology, Institute of Pathology, Ludwig-Maximilians-Universität München, Munich, Germany; 2 Institute of Pathology, Ludwig-Maximilians-Universität München, Munich, Germany; 3 German Cancer Consortium (DKTK), Partner site Munich, Munich, Germany; 4 German Cancer Research Center (DKFZ), Heidelberg, Germany

## Abstract

Epithelial-mesenchymal transition **(**EMT) plays an important role in tumor invasion and metastasis. A comprehensive, bioinformatics analysis of CCLE and TCGA datasets of seven tumor types allowed us to identify a novel pan-cancer EMT-associated gene expression signature consisting of 16 epithelial and 4 mesenchymal state-associated mRNAs. Among the identified epithelial cell state-associated factors, down-regulation of the *RBM47* (RNA binding motif protein 47) mRNA displayed the most significant association with metastasis and poor survival in multiple cohorts of colorectal cancer (CRC) patients. Moreover, decreased RBM47 protein expression was associated with metastasis in a cohort of primary CRCs. *RBM47* was directly suppressed during EMT induced by IL6-activated STAT3 or ectopic SNAIL and SLUG expression via conserved binding motifs of these factors within the *RBM47* promoter. Moreover, RNAi-mediated down-regulation of RBM47 in CRC lines resulted in increased cell migration, invasion and metastases formation. As demonstrated by the example of RBM47, the EMT-associated signature characterized here allows to identify biomarkers for predicting clinical outcome of CRC and presumably other cancer entities. In addition, our functional analysis of RBM47 shows that the down-regulation of RBM47 during CRC progression may promote EMT and metastasis.

## Introduction

Metastasis accounts for over 90% of cancer mortality^1^. A better understanding of the molecular mechanism of metastasis holds the promise of providing biomarkers and therapeutic strategies to overcome this situation. During the metastatic process epithelial tumor cells acquire a migratory mesenchymal phenotype, which allows them to leave the site of the primary tumor, invade surrounding tissues and migrate to distant organs^2, 3^. The process by which cells switch from an epithelial to a mesenchymal phenotype is known as the epithelial-mesenchymal transition (EMT)^4^. A hallmark of EMT is the loss of the cell-cell adhesion protein E-Cadherin (encoded by the *CDH1* gene) and gain of the cytoskeletal protein Vimentin (encoded by the *VIM* gene)^5^. In recent years, several factors that regulate EMT have been identified, with SNAIL (SNAI1), SLUG (SNAI2), TWIST1, ZEB1, and ZEB2 representing the core EMT-TFs (EMT-inducing transcription factors) that also mediate EMT during development^6, 7^. These EMT-TFs directly regulate the expression of *E-cadherin* or *Vimentin* ^8^. Many additional EMT-inducing factors have been implicated in cancer progression and represent potential diagnostic markers or targets for cancer therapy^6, 9–11^. In addition, several EMT-associated gene signatures have been identified^12–17^. Recently, a consensus molecular subtype (CMS) classification was proposed which subdivides CRCs into four main groups^18^. Notably, cancers that belong to the CMS4 subtype, which is associated with high expression of mesenchymal genes and low expression of epithelial genes, exhibit the worst prognosis.

However, the link between EMT and tumor progression is far from being completely understood. To identify novel EMT-associated markers in cancers, we performed a combined bioinformatic analysis of expression data from The Cancer Genome Atlas (TCGA)^19^ and Cancer Cell Line Encyclopedia (CCLE)^20^ databases. Only markers differentially regulated in both, cancer cell lines and primary tumors, were considered for further analysis. Thereby, we identified a novel pan-cancer EMT-associated gene signature consisting of 16 epithelial and 4 mesenchymal state-associated markers. Since the TCGA expression profiles were obtained from whole tumors, stromal cells might lead to the false detection of mesenchymal markers indicative of EMT as they show a markedly higher expression in stromal cells than in cancer cells^21–23^. Because of the problematic nature of mesenchymal tumor markers we focused on the analysis of epithelial markers. We performed an exemplary validation of the RNA binding motif protein 47 (RBM47) as an epithelial state-associated factor and found that it is required for the maintenance of an epithelial state and the suppression of migration, invasion, and metastasis. Moreover, analysis of patient samples showed that down-regulation of RBM47 protein expression represents a potential marker for CRC progression.

## Results

**Figure 1:**
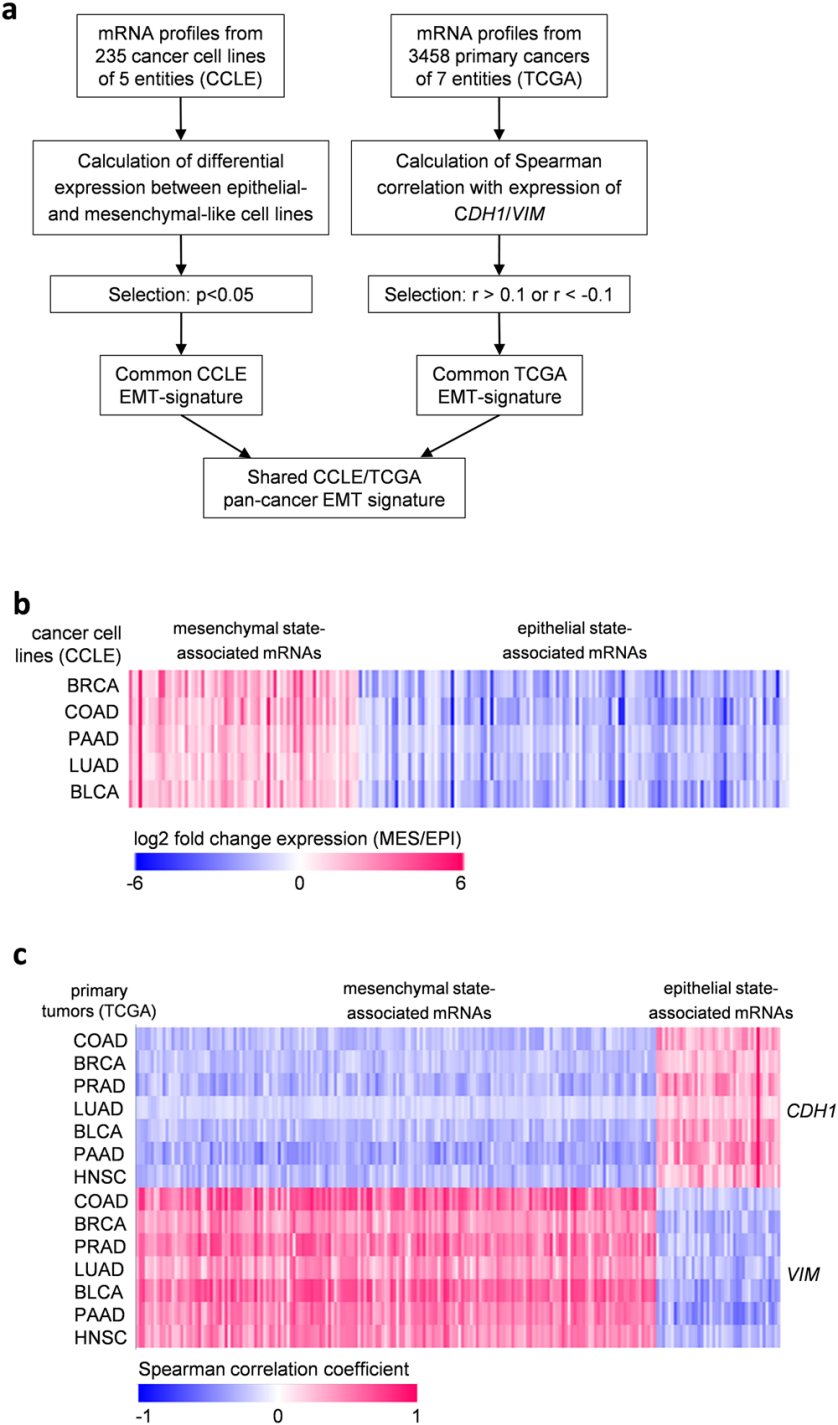
Bioinformatic analysis of CCLE and TCGA datasets for EMT-associated signatures. (**a**) Flow chart describing the generation of the shared pan-cancer CCLE/TCGA EMT-associated mRNA signature. (**b**) Heatmap of 201 mRNAs that showed significant differences in expression between epithelial-like and mesenchymal-like cancer cell lines within the analysed cancer entities (log2 fold change of expression is shown; p < 0.05). (**c**) Heatmap showing Spearman correlation coefficients of 365 epithelial and mesenchymal state-associated mRNAs with *CDH1* and *VIM*. Epithelial state-associated mRNAs were defined as mRNAs that positively correlate with expression of the epithelial marker *CDH1* (r > 0.1) and negatively correlate with expression of the mesenchymal marker *VIM* (r < −0.1) in all indicated TCGA datasets. Mesenchymal state-associated mRNAs were defined as mRNAs that negatively correlate with expression of the epithelial marker *CDH1* (r < −0.1) and positively correlate with expression of the mesenchymal marker *VIM* (r > 0.1) in all indicated TCGA datasets. COAD, colon cancer; BRCA, breast cancer; LUAD, lung cancer; HNSC, head&neck squamous cel cancer; PRAD, prostate adenocarcinomas; BLCA, bladder cancer; PAAD, pancreatic adenocarcinomas.

To identify novel putative EMT-associated genes, we performed a combinatorial bioinformatics analysis of CCLE and TCGA public databases (Fig. 1a). First, we utilized expression data from the CCLE database^20^, which contains mRNA expression profiles of approximately 1000 cancer cell lines representing 37 cancer entities. We grouped cell lines from colorectal, breast, lung, bladder, and pancreatic cancer based on their epithelial/mesenchymal characteristics: epithelial-like cell lines (expression of *CDH1* more than 10-fold higher than expression of *VIM*) and mesenchymal-like cell lines (expression of *VIM* more than 10-fold higher than expression of *CDH1*) (Supplemental Fig. S1a; example for CRC cell lines provided). Next, we compared the expression of all mRNAs between epithelial-like and mesenchymal-like cancer cell lines (Fig. 1b). 131 mRNAs showed a significantly higher expression in epithelial-like cell lines compared to mesenchymal-like cell lines from all analyzed cancer entities, whereas 70 mRNAs were significantly lower expressed in epithelial-like cell lines (Fig. 1b, Table 1, and Supplemental Data 1). Next, we performed a second screen by using next generation sequencing RNA expression data of seven cancer entities from TCGA^19^. Thereby, we determined which mRNAs present in the TCGA datasets show a negative correlation with the mesenchymal marker *VIM* and positive correlation with the epithelial marker *CDH1* (epithelial-state associated mRNAs) or positive correlation with the mesenchymal marker *VIM* and negative correlation with the epithelial marker *CDH1* (mesenchymal-state associated mRNAs) (Fig. 1c and Supplemental Fig. S1b). To identify factors that are generally associated with EMT and therefore presumably of functional importance for EMT in the analyzed TCGA datasets, we selected those mRNAs that are shared in all analyzed cancer entities and obtained a list of 42 epithelial state-associated mRNAs and 323 mesenchymal state-associated mRNAs (Fig. 1c, Table 1, and Supplemental Data 2). Finally, 16 epithelial state-associated mRNAs and 4 mesenchymal state-associated mRNAs were shared between the CCLE and TCGA screens (Table 1 and Supplemental Fig. S2). The TCGA expression data was obtained from whole tumors. Therefore, stromal cells within tumors might contribute to the overall expression of a gene. This is especially important for mesenchymal-state associated genes, which commonly show one or two orders of magnitude higher expression in stromal cells than in cancer cells^21–23^. On contrary, CCLE expression data is derived from cancer cell lines in the absence of tumor stroma. This might explain the low concordance between TCGA and CCLE derived mesenchymal state-associated mRNAs.

**Table 1:** Identification of mRNAs associated with epithelial- or mesenchymal-state as determined by analysis of CCLE and TCGA datasets derived from indicated cancer entities.

Cancer entity	Number of samples		Number of epithelial state-associated mRNAs		Number of mesenchymal state-associated mRNAs	
	CCLE	TCGA	CCLE	TCGA	CCLE	TCGA
Colon adenocarcinoma	46	462	1311	2212	3247	2864
Breast invasive carcinoma	40	1095	3454	2604	2478	4485
Lung adenocarcinoma	106	515	2954	653	2075	1156
Pancreatic adenocarcinoma	21	178	754	3292	468	5344
Bladder urothelial carcinoma	22	408	969	3787	1815	4156
Prostate adenocarcinoma	n.a.	497	n.a.	2888	n.a.	3945
Head&Neck squamous cell cancer	n.a.	303	n.a.	2077	n.a.	4140
Shared EMT-associated mRNAs identified in all analyzed cancer types			131	42	70	323
Shared CCLE/TCGA EMT-associated genes			16		4	

In the CCLE screen, epithelial-state associated mRNAs were defined as mRNAs that showed a significantly higher expression in epithelial-like cancer cell lines compared with mesenchymal-like cell lines. Mesenchymal-state associated mRNAs were defined as mRNAs that showed a significantly higher expression in mesenchymal-like cancer cell lines compared with epithelial-like cell lines. Significance was calculated by a Student’s t-test (p < 0.05 was considered significant). n.a., not analyzed. In the TCGA screen, epithelial-state associated mRNAs were defined as mRNAs that positively correlated with expression of the epithelial marker *CDH1* (r > 0.1) and negatively correlated with expression of the mesenchymal marker *VIM* (r < −0.1). Mesenchymal-state associated mRNAs were defined as mRNAs that negatively correlated with expression of the epithelial marker *CDH1* (r < −0.1) and positively correlated with expression of the mesenchymal marker *VIM* (r > 0.1).

**Figure 2:**
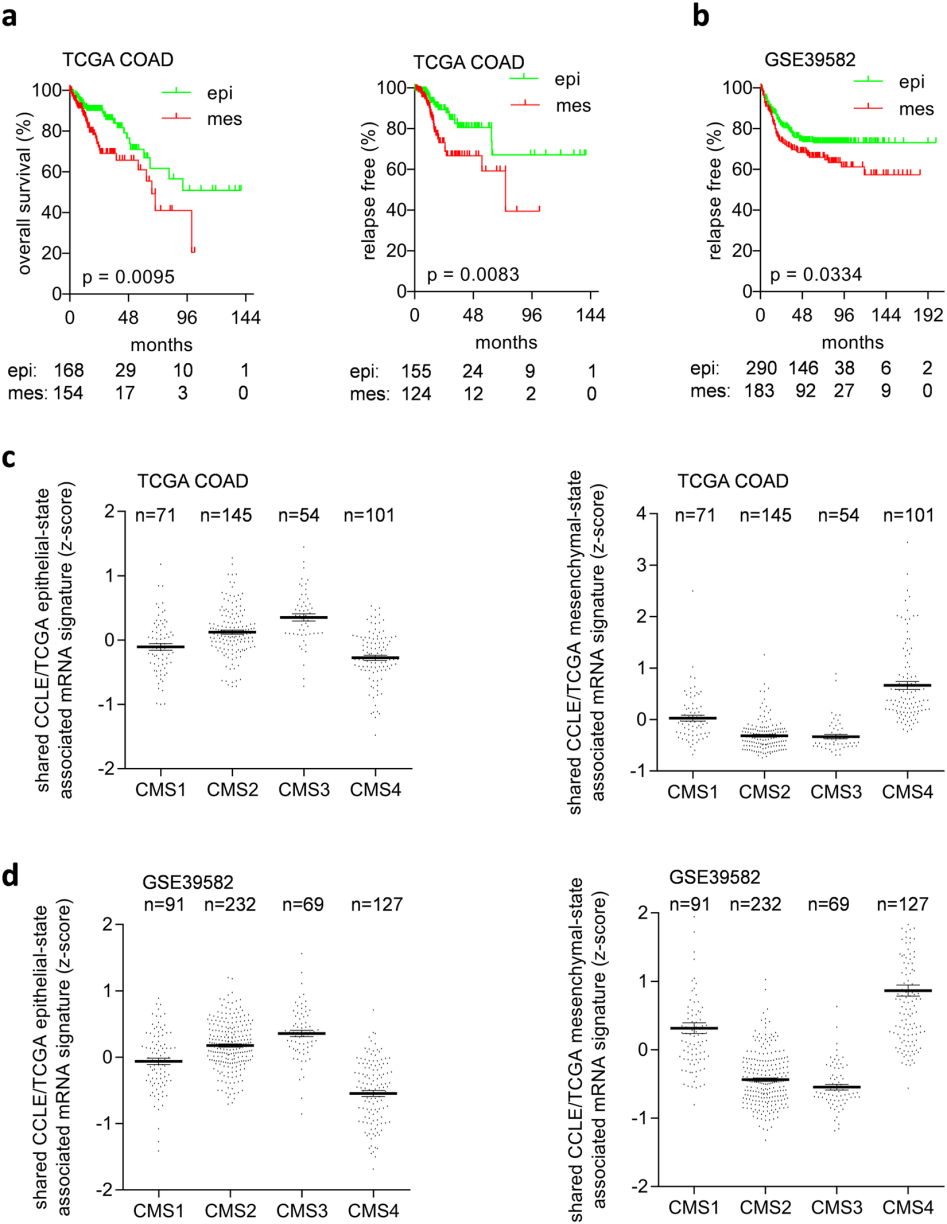
EMT-associated mRNA signatures are associated with survival in colon cancer patients. (**a,b**) Associations of epithelial state (epi) and mesenchymal state (mes) associated groups of samples with survival in TCGA COAD (**a**) and the GSE39582 datasets (**b**) (epi and mes groups of samples were defined by K-means clustering based on expression of the shared CCLE/TCGA epithelial state- or mesenchymal state-associated mRNAs. (**c,d**) Association of the shared TCGA/CCLE epithelial state- and mesenchymal state-associated mRNA signatures with the consensus molecular subtypes (CMS) of colorectal cancer in TCGA (**c**) and GSE39582 datasets (**d**).

The shared CCLE/TCGA epithelial- and mesenchymal state-associated gene signatures were used to cluster samples from the TCGA COAD and GSE39582 cohorts in two groups by k-means clustering. In both cohorts, patients with high expression of the mesenchymal-like mRNA signature had a worse overall and relapse-free survival than patients from the epithelial-associated group (Fig. 2a and b). Comparison of the identified EMT-associated signatures with published EMT-signatures showed some overlap (Supplemental Table S1). Comparison with the CMS classification system showed lowest expression of the shared TCGA/CCLE epithelial state-associated signature in the mesenchymal CMS4 subtype in two independent CRC patient cohorts (Fig. 2c and d), whereas the shared TCGA/CCLE mesenchymal state-associated signature showed the highest expression in the mesenchymal CMS4 subtype (Fig. 2c and d).

**Figure 3:**
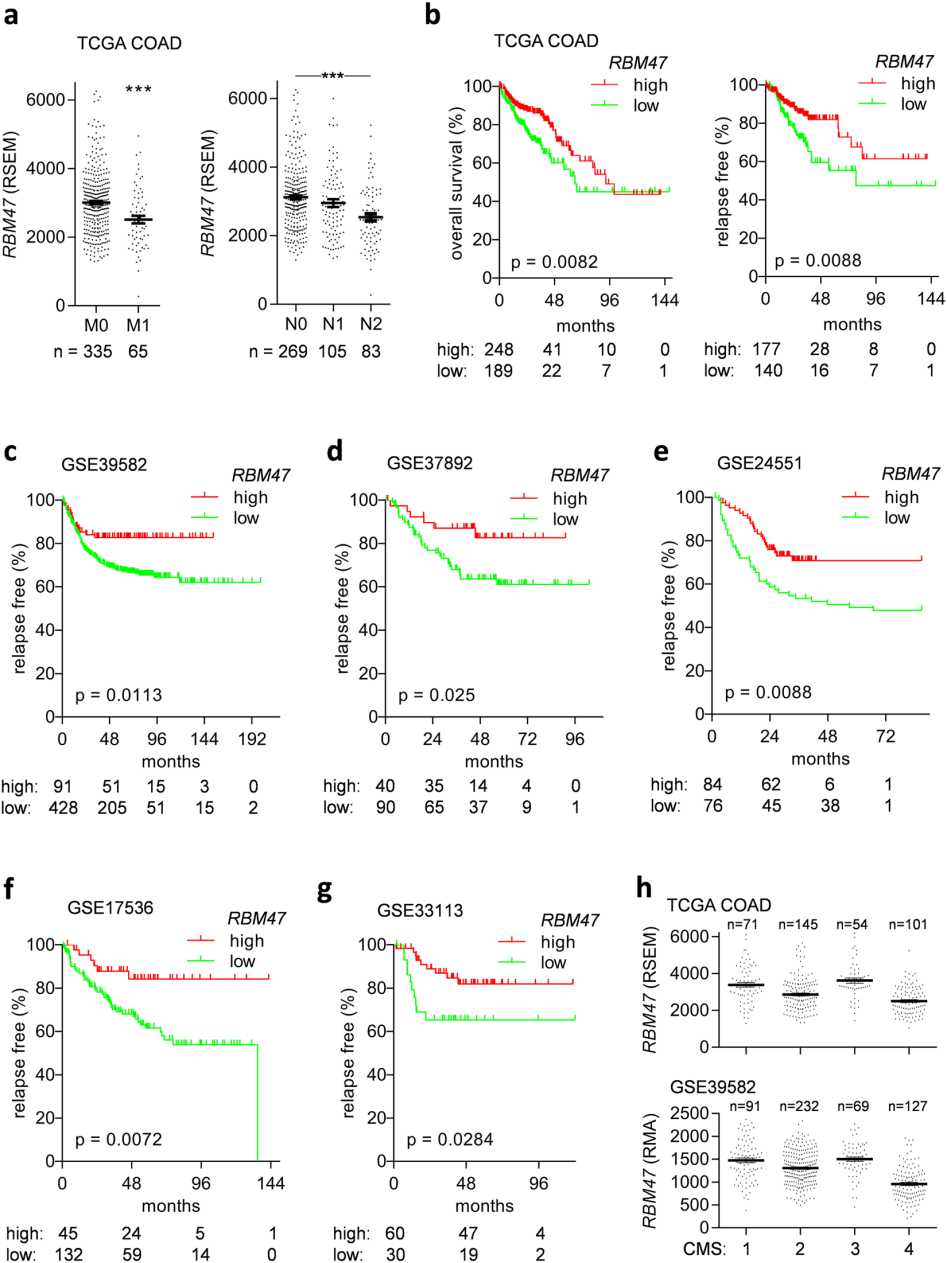
Low expression of *RBM47* mRNA is associated with metastasis, nodal status, and poor survival in CRC patients. (**a**) Association of *RBM47* mRNA expression with metastasis (right) and nodal status (left) in the TCGA COAD dataset. (**b**) Association of *RBM47* mRNA expression with overall and relapse free survival in the TCGA COAD dataset. (**c**–**g**) Associations of *RBM47* mRNA expression with relapse free survival in indicated CRC dataset. (**h**) Association of *RBM47* mRNA expression with the consensus molecular subtypes (CMS) of colorectal cancer in TCGA and GSE39582 datasets.

Several of the EMT-associated genes identified here, such as *ESRP1* and *ESRP2*, have well established roles in EMT^24, 25^, indicating that the bioinformatics screen applied here was appropriate for identifying functionally relevant regulators of EMT. Next, we analyzed whether the identified EMT-associated mRNAs associate with nodal status and distal metastasis in the TCGA COAD cohort (Table 2). The most significant association with these clinical parameters was observed for the *RBM47* mRNA, encoding the RNA binding motif 47. Notably, decreased expression of the *RBM47* mRNA was also significantly associated with presence of distal metastases and increased nodal status in the TCGA COAD cohort (Fig. 3a). Moreover, low *RBM47* expression was significantly associated with poor overall and relapse free survival in TCGA COAD and five additional CRC patient cohorts (Fig. 3b–g), Decreased expression of *RBM47* was also significantly associated with poor overall and relapse free survival in the TCGA pan-cancer dataset, which encompasses expression profiles of more than 8400 cancer patients from 30 different entities (Supplemental Fig. S3). Finally, the expression of *RBM47* was lowest in the mesenchymal CMS4 subtype in two independent CRC patient cohorts (Fig. 3h).

**Table 2:** Shared TCGA/CCLE epithelial state- and mesenchymal state-associated mRNAs and their associations with distant metastasis and nodal status in the TCGA COAD cohort.

Shared TCGA/CCLE epithelial state-associated mRNAs	Fold change M0/M1	Fold change N0/N+	Shared TCGA/CCLE mesenchymal state-associated mRNAs	Fold change M0/M1	Fold change N0/N+
*AFTPH*	1.04	1.01	*LGALS1*	1.35*	1.23
*CDH1*	1	1.04	*P4HA3*	1.3	1.34*
*CDS1*	0.89^*^	0.90^**^	*UROD*	0.95	1.03
*EPCAM*	1.02	1.05	*VIM*	1.41*	1.33*
*ESRP1*	1.02	1.02			
*ESRP2*	1.04	1.00			
*FA2H*	0.93	1.02			
*HOOK1*	0.94	0.91^*^			
*LNX1*	1.00	1.06			
*MAP7*	1.01	0.94			
*MAPK13*	1.03	1.06			
*MARVELD2*	1.03	0.99			
*MARVELD3*	0.88^*^	1.00			
*MYO5B*	0.90^*^	0.92^*^			
*MYO5C*	0.83^**^	0.85^***^			
*RBM47*	0.84^***^	0.89^***^			

Fold change: average expression of indicated mRNAs in primary tumors from patients without distant metastases (M0) or negative nodal status (N0) divided by average expression of indicated mRNAs in primary tumors from patients with distant metastases (M1) or positive nodal status (N+). *P < 0.05; **P < 0.01; ***P < 0.001.

**Figure 4:**
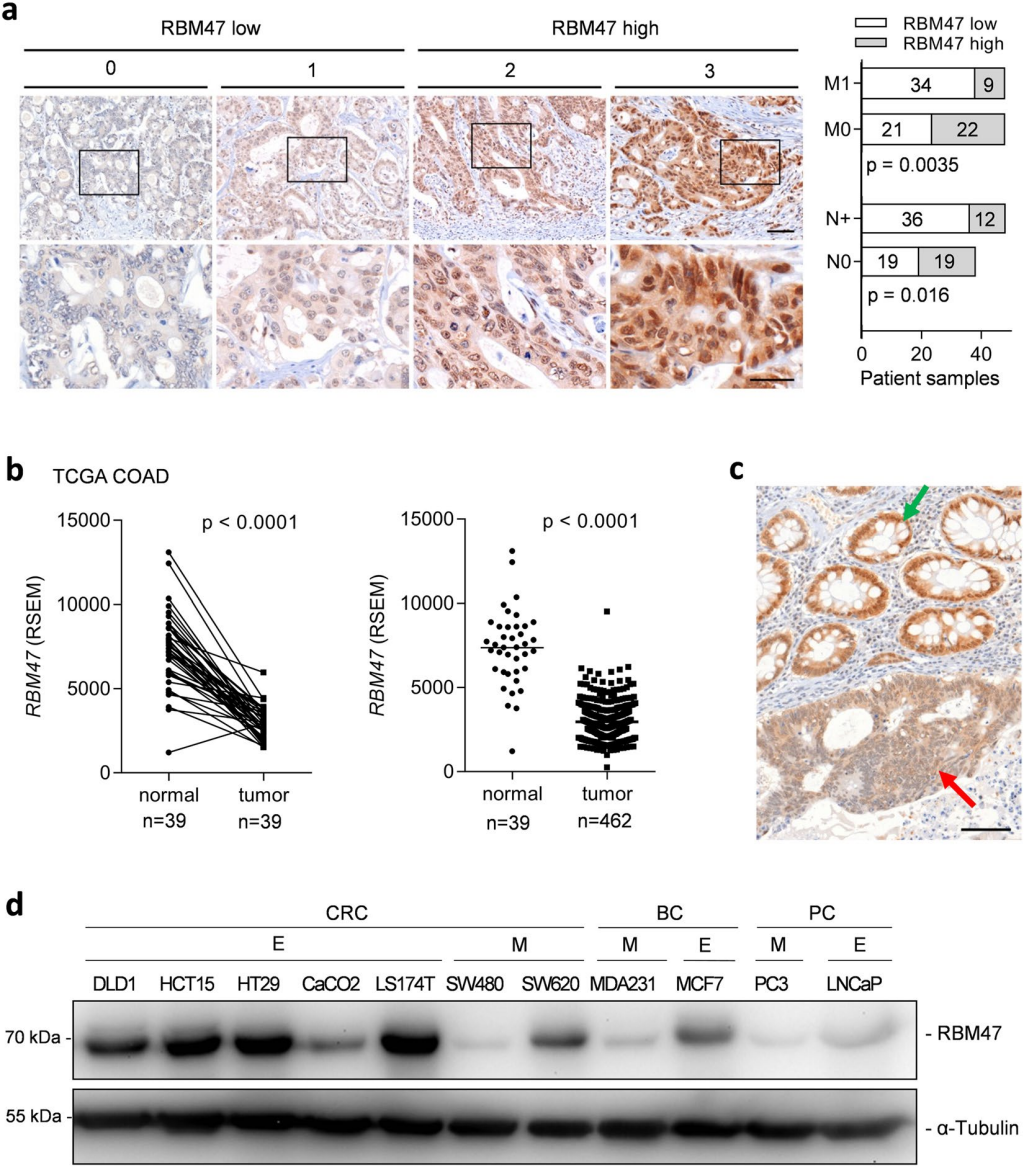
Low expression of RBM47 protein is associated with metastasis and positive nodal status in CRC patients (**a**) Association of RBM47 protein expression with liver metastasis (M1) and nodal metastasis (N+) in the M0/M1 patient collection (N = 86). Left panel: representative IHC staining results of patient samples with staining scores. Right panel: quantification of IHC staining and association with metastasis (M0 – no metastases present, M1 – liver metastases present) and nodal status (N0 – no tumor cells present in lymph nodes, N+ - tumor cells present in lymph nodes). The scale bar in low magnification images (upper part) represents 100 μm and the bar in high magnification images (lower part) represents 50 μm. (**b**) Expression of *RBM47* mRNA in normal colonic tissue and colon tumors in the TCGA COAD dataset. In the left panel expression of RBM47 in paired samples from the same patient are shown and in the right panel all samples are shown. (**c**) Exemplary IHC results showing expression of RBM47 protein in a colon cancer (red arrow) and adjacent normal colonic mucosa (green arrow). The scale bar represents 100 μm. (**d**) Immunoblot analysis of RBM47 expression in a cell line panel consisting of epithelial-like (E) and mesenchymal-like (M) colorectal (CRC), breast (BC), and prostate (PC) cancer cell lines.

Next we determined the expression of RBM47 protein in a matched case control cohort of primary CRCs with (n = 43) and without (n = 43) liver metastasis (Supplemental Table S2). Patients with liver metastases expressed lower levels of RBM47 in primary CRCs (Fig. 4a), suggesting that loss of RBM47 may promote metastasis. Additionally, significantly more patients with positive nodal status showed low RBM47 expression when compared to patients with nodal status N0 (Fig. 4a). Interestingly, the expression of *RBM47* mRNA was also lower in CRC tumors when compared with adjacent normal colonic tissue in samples from TCGA COAD cohort (Fig. 4b). Likewise, immuno-histochemical analysis of 31 CRC samples exhibiting adjacent normal mucosa showed that in the majority of cases RBM47 protein expression was higher in normal colonic mucosa than in the adjacent tumor tissue (see representative example in Fig. 4c and Supplemental Table S3). In line with these results obtained in patient derived samples the expression of RBM47 was elevated in epithelial-like cell lines and decreased in mesenchymal-like cell lines derived from colorectal, breast, and prostate cancer (Fig. 4d). The epithelial-like or mesenchymal-like phenotype of the cell lines analyzed here has been determined previously^26^. Taken together, these results demonstrate that down-regulation RBM47 is highly associated with tumor progression and EMT.

**Figure 5:**
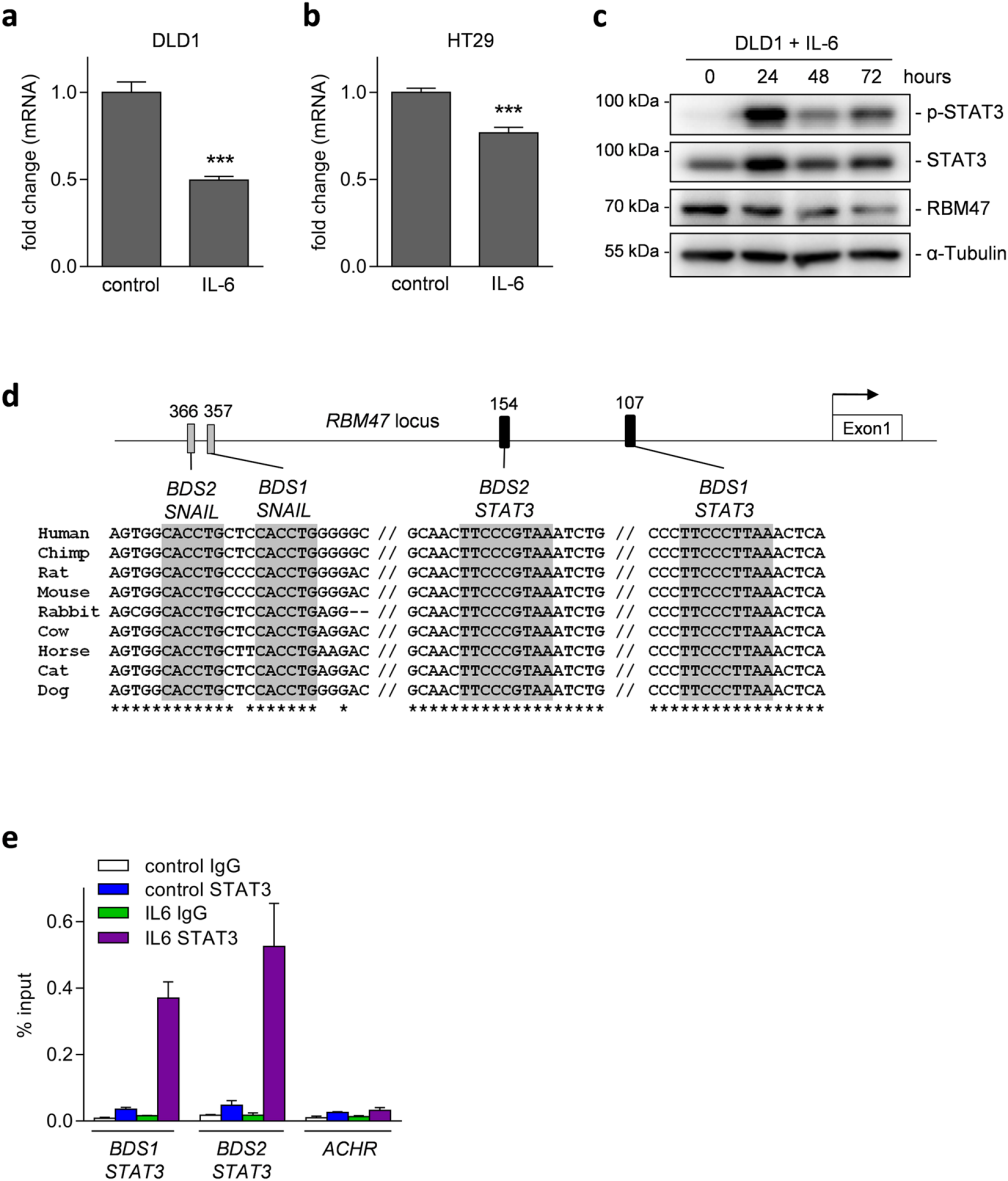
Direct suppression of *RBM47* by IL-6 activated STAT3. (**a** and **b**) Expression of *RBM47* mRNA in (**a**) DLD1 or (**b**) HT29 CRC cells after treatment with IL-6 (20 ng/ml) or vehicle for 72 hours. (**c**) Western blot analysis of indicated proteins in DLD1 cells after exposure to IL-6 (20 ng/ml) for the indicated periods. (**d**) Map of the human *RBM47* promoter region with the indicated conserved STAT3 and SNAIL binding sites. Filled rectangles represent the binding sites. TF binding sequence motifs are indicated by grey shadowing. Their conservation between species is indicated by asterisks. The arrow indicates the TSS, // represent additional, not shown sequences between the BDS. (**e**) qChIP analysis of STAT3 occupancy at the *RBM47* promoter and, as a control, the acetylcholine receptor (*ACHR*) locus in DLD1 cells treated with vehicle or IL-6 for 20 minutes. In a,b, and e mean values ± SD (n = 3) are provided with *P < 0.05; **P < 0.01; ***P < 0.001.

In order to identify up-stream regulators and oncogenic signaling pathways that may cause the observed down-regulation of *RBM47* in mesenchymal-like cancer cells during tumor progression, we analyzed *RBM47* expression after experimental induction of EMT in CRC cell lines. We have previously shown that treatment of epithelial-like DLD1 CRC cells with interleukin-6 (IL-6) induces EMT^26^. Importantly, the tumor microenvironment as well as tumor cells are known to produce IL-6, which enhances tumor progression^27, 28^. Notably, expression of *RBM47* mRNA was repressed in DLD1 and HT29 CRC cells upon treatment with IL-6 for 72 hours (Fig. 5a and b). Also on the protein level RBM47 was repressed by IL-6 treatment which coincided with activation of the STAT3 transcription factor (Fig. 5c). Inspection of the *RBM47* promoter region revealed two potential binding sites for STAT3, 107 (BDS1) and 154 (BDS2) base pairs (bp) upstream of the TSS (transcriptional start site) (Fig. 5d). Indeed, we could verify the binding of STAT3 to both sites by quantitative chromatin immune-precipitation (qChIP): STAT3 binding was enriched at both predicted binding sites in IL-6 treated DLD-1 cells, whereas no significant enrichment was observed in the absence of IL-6 (Fig. 5e). Therefore, RBM47 expression is under direct, negative control of the IL-6/IL-6R/STAT3 pathway.

**Figure 6:**
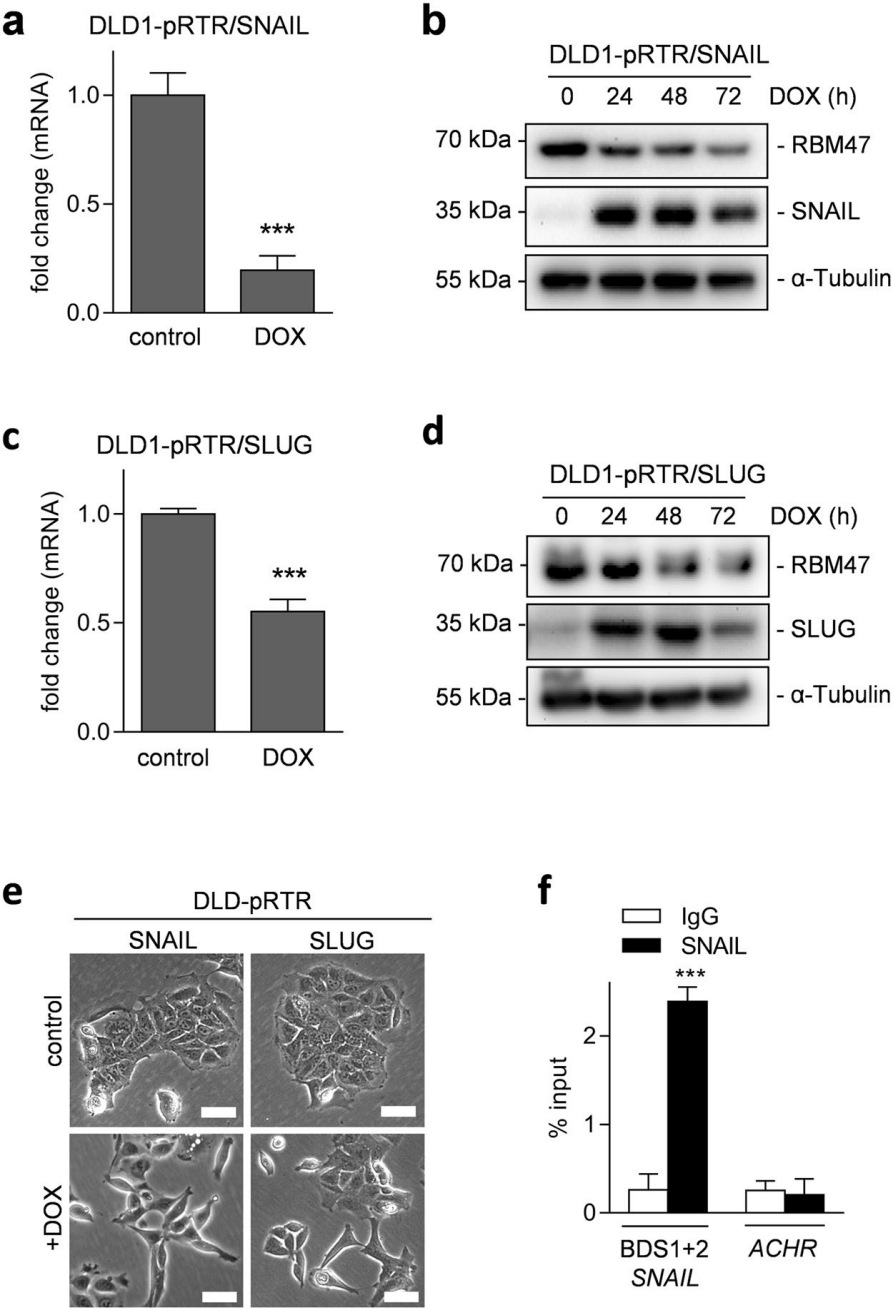
Direct suppression of *RBM47* by the EMT-TFs SNAIL and SLUG. (**a**) qPCR analysis of *RBM47* mRNA and (**b**) Western blot analysis of the indicated proteins in DLD1 cell pools harboring pRTR/SNAIL plasmids. In (**a**) cells were treated with DOX or vehicle for 72 hours, in (**b**) with DOX for the indicated periods. (**c** and **d**) as in (**a**) and (**b**) but using DLD1 cell pools harboring pRTR/SLUG plasmids. (**e**) Phase contrast microscopy of the DLD-pRTR/SNAIL and DLD-pRTR/SLUG cell lines treated with vehicle or DOX for 48 hours. Scale bars represent 50 μm. (**f**) qChIP analysis of SNAIL occupancy at the *RBM47* promoter and, as a control, the acetylcholine receptor (*ACHR*) locus in SW480 cells. In a, c, and f mean values ± SD (n = 3) are provided. *P < 0.05; **P < 0.01; ***P < 0.001.

When inspecting the *RBM47* promoter we also noticed two adjacent canonical SNAIL binding sites 357 bp and 366 bp upstream of the TSS (Fig. 5d). Therefore, we analyzed whether ectopic expression of SNAIL or SLUG, which represent canonical EMT-TFs, suppresses *RBM47*. Indeed, activation of conditional *SNAIL* and *SLUG* alleles suppressed expression of RBM47 at the mRNA and protein levels in DLD1 CRC cells (Fig. 6a–d). Ectopic expression of SNAIL or SLUG resulted in morphologic changes characteristic for EMT: A tightly packed, cobble-stone like growth pattern converted into a scattered pattern with cells adopting a spindle-like shape (Fig. 6e). Next, we analyzed the binding of SNAIL to the *RBM47* promoter in SW480 CRC cells, which express high levels of SNAIL^11^. qChIP analysis showed an enrichment of SNAIL at the potential SNAIL binding sites in the *RBM47* promoter (Fig. 6f). Therefore, *RBM47* is also repressed by EMT-TFs, which are up-regulated in cancer cells during tumor progression and mediate EMT, thereby promoting invasion and presumably metastasis.

**Figure 7:**
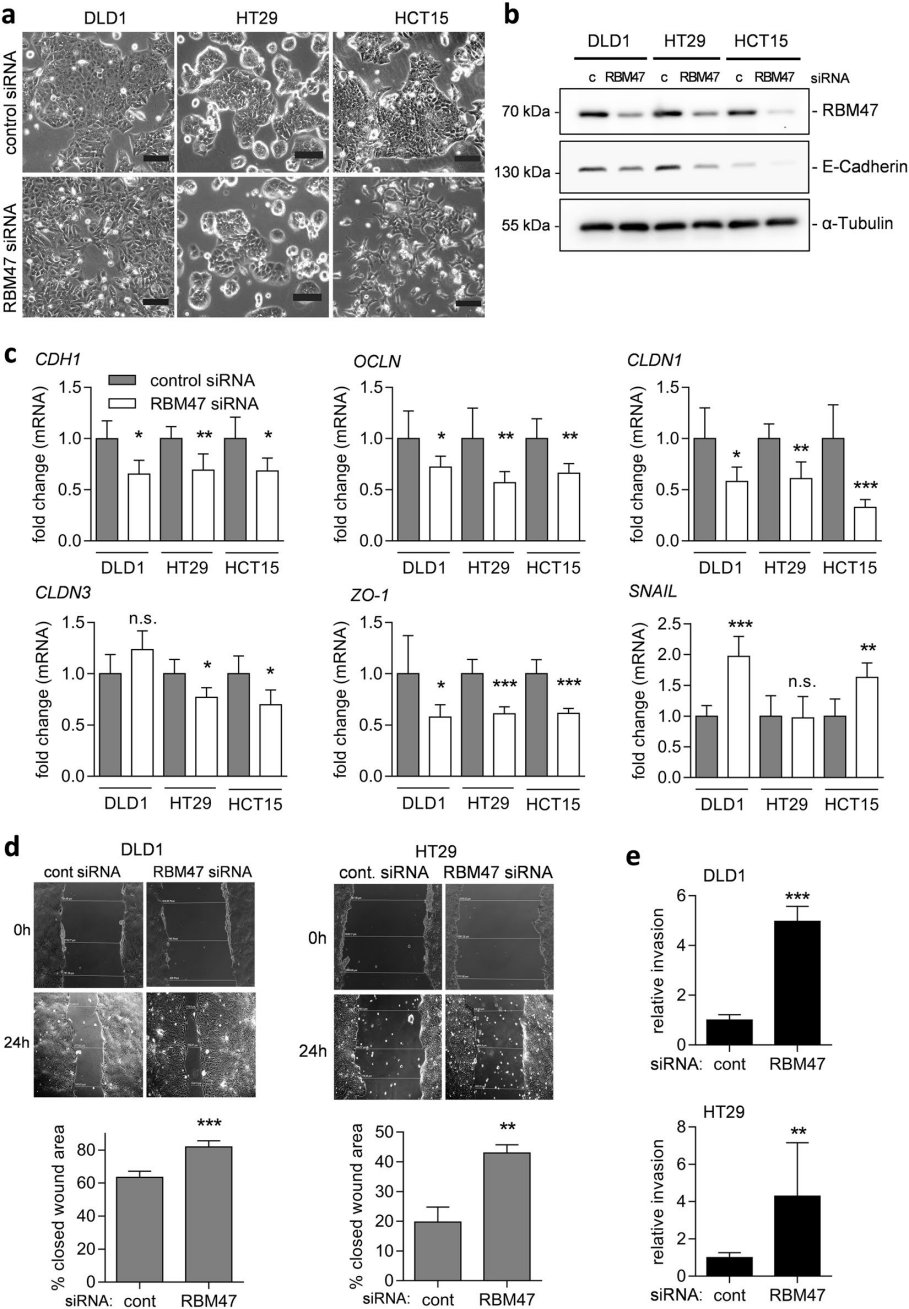
Inhibition of *RBM47* induces EMT, cell migration and invasion. (**a**) Phase contrast microscopy of the indicated CRC cell lines transfected with control or *RBM47*-specific siRNA. Scale bars represent 100 μm. (**b**) Western blot analysis of the indicated proteins in CRC cell lines transfected with control or *RBM47*-specific siRNA for 72 h. (**c**) qPCR analysis of the indicated mRNAs in the indicated CRC cell lines transfected with control or *RBM47*-specific siRNA for 72 h. (**d**) Wound healing assay of the indicated CRC cell lines transfected with control or *RBM47*-specific siRNA 48 hours before a scratch was generated. (Upper panels) Representative photographs of the initial wound area and the same area 24 hours later. (Lower panels) Quantification of wound closures: The width of scratches in 2 independent wells was analyzed for each state. Results represent the average (%) of wound closure. (**e**) Relative invasion of indicated CRC cells in matrigel-coated Boyden chambers transfected with control or RBM47-specific siRNA for 48 hours. In c, d, and e mean values ± SD (n = 3) are provided. *P < 0.05; **P < 0.01; *** P < 0.001.

**Figure 8:**
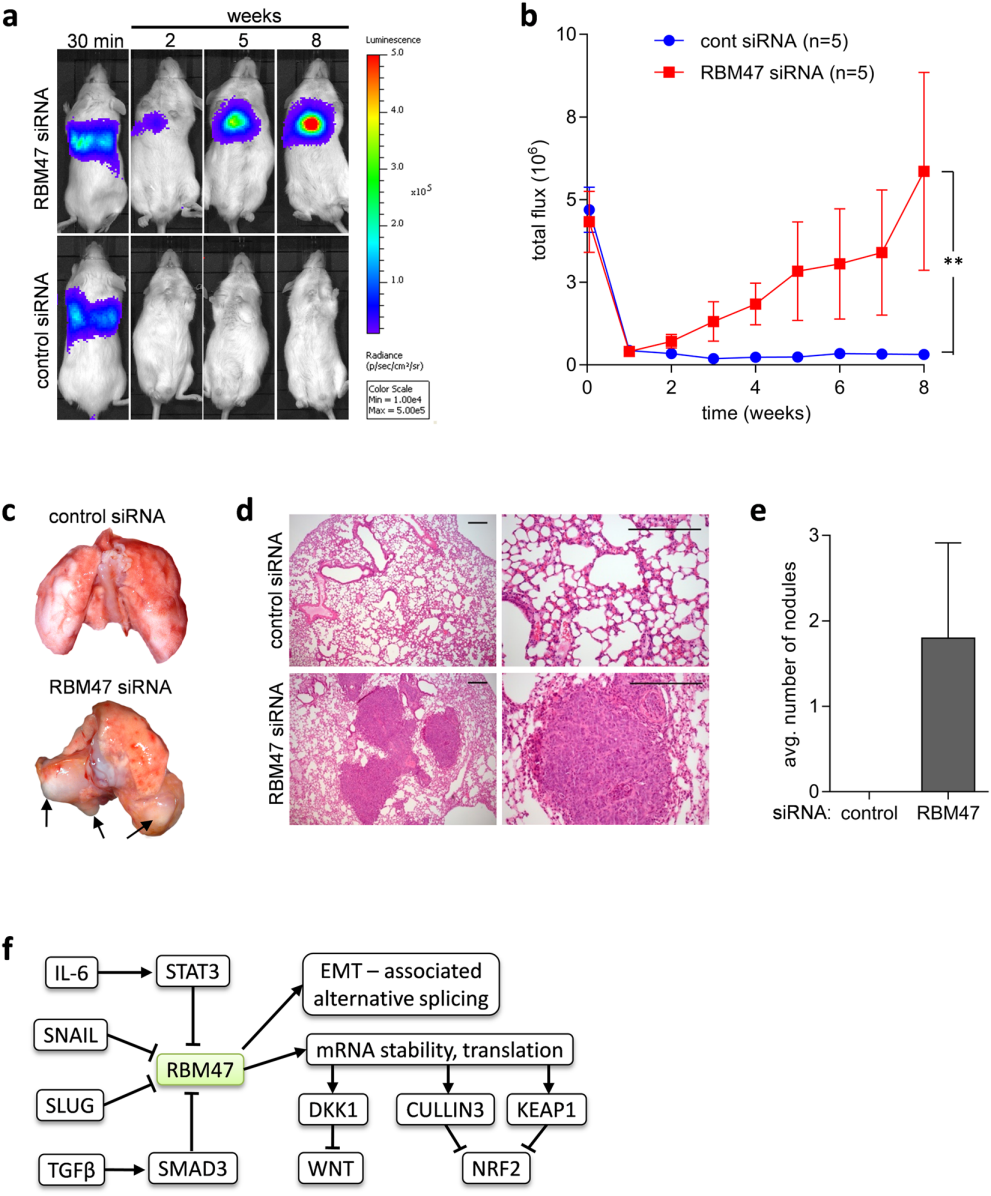
Inhibition of *RBM47* induces metastasis. Formation of lung metastases after tail-vein injection of DLD-1–Luc2 cells transfected with control or *RBM47*-specific siRNA. (**a**) Representative images of luciferase signals after D-luciferin injection at the indicated time points after xenografting. (**b**) Weekly measurements of total photon flux. Results are the mean ± SD (n = 5). **P < 0.01. (**c**) Representative lungs 8 weeks after tail vein injection. (**d**) H&E staining of lung tissue. Scale bars represent 500 μm. (**e**) Number of metastatic nodules in lungs 8 weeks after tail vein injection of the indicated DLD1 cells into mice (n = 5). (**f**) Model of regulation of RBM47 expression and its downstream targets, which presumably mediate its effects on EMT and cancer progression.

To investigate whether the down-regulation of RBM47 induces EMT, tumor invasion, and metastasis, *RBM47* was silenced by *RBM47*-specific siRNAs in DLD1, HCT15, and HT29 CRC cell lines. In HCT15 and partially in DLD1 cells suppression of *RBM47* resulted in a transition from an epithelial to a mesenchymal morphology: tightly packed, cobble-stone like cells became spindle-shaped cells with a scattered growth pattern, whereas in HT29 cells no change in morphology was observed (Fig. 7a). Furthermore, silencing of *RBM47* in DLD1, HT29, and HCT15 CRC cells resulted in repression of the epithelial-associated mRNAs *E-cadherin* (*CDH1*), *OCLN*, *CLDN1*, *CLDN3*, and *ZO-1*, as well as induction of the EMT-inducer *SNAIL* in DLD1 and HCT15 cells (Fig. 7b and c). The lack of SNAIL induction in HT29 cells might explain the absence of changes in cell morphology of these cells after RBM47 knockdown. EMT has been previously associated with increased cancer cell migration, invasion and metastasis^7^. Accordingly, siRNA-mediated silencing of *RBM47* in DLD1 and HT29 cells increased cell migration in wound healing assays and invasion in modified Boyden chamber assays (Fig. 7d and e). Finally, DLD1-luc2 cells transfected with *RBM47*-specific siRNAs formed lung metastases when injected into tail veins of immune compromised mice, whereas no metastases formation was observed with non-invasive imaging when DLD1-luc2 cells transfected with control siRNAs were injected (Fig. 8a and b). After resection of the lungs eight weeks after injection, macroscopically visible metastases were detectable in mice, which had received *RBM47*-specific siRNA treated cells, whereas mice injected with cells treated with control siRNA did not show any visible metastases (Fig. 8c). Haematoxylin and eosin (H&E) staining revealed the presence of metastatic nodules in resected lungs of mice injected with cells transfected with *RBM47*-specific siRNAs, whereas no metastases were detectable in lungs of mice injected with cells transfected with control siRNAs (Fig. 8d and e). Taken together, these results demonstrate that the loss of RBM47 induces EMT and promotes migration, invasion, and metastases formation of CRC cells (for a summarizing model see Fig. 8f).

## Discussion

By utilizing expression data from online cancer databases, we performed a bioinformatic screen to identify novel EMT-associated mRNA markers. Thereby, we identified a novel pan-cancer EMT-associated signature. The markers identified by analysis of the CCLE cancer cell line data clearly reflect cancer-associated EMT, since they are derived from cancer cells only. However, in TCGA expression profiles, which are derived from bulk tumors, mesenchymal state-associated mRNAs may also be derived from stromal cells. Since mesenchymal markers are expressed up to 10 fold higher in stromal cells, even a small fraction of stromal cells can generate false positive mesenchymal tumor markers that may be misinterpreted as indicating EMT of tumor cells. However, epithelial state-associated mRNAs are predominantly expressed by cancer cells and thus varying fractions of stromal components will not affect detection of their expression^21–23^. In this study we therefore focused on epithelial state-associated mRNAs, which are down-regulated in CRCs and in CRC cell lines that had undergone EMT. Several of the identified epithelial state-associated mRNAs, such as *EPCAM*, *ESRP1*, and *ESRP2*, have well established roles in EMT^24, 25, 29^ indicating that the bioinformatics approach used here was appropriate. However, we also identified differential expression of genes, such as *CDS1*, *MAP7*, and *RBM47*, for which an involvement in EMT was relatively unknown. Because of its strong association with positive nodal status and distant metastasis we focused on RBM47 in order to functionally validate an exemplary candidate obtained by this approach. By analyzing mouse models and patient collections we could show that down-regulation of RBM47 promotes colorectal cancer metastasis and is associated with poor survival of CRC patients. In support of our findings, *RBM47* was previously reported as one of the genes down-regulated genes in the mesenchymal CRC sub-type associated with poor survival^30^. Furthermore, we showed that RBM47 is suppressed during CRC-associated EMT that was triggered by several different stimuli. Moreover, suppression of RBM47 enhanced CRC cell migration, invasion, and metastasis, suggesting that inactivation of RBM47 function promotes CRC progression. We also showed that elevated expression of RBM47 is strongly associated with the epithelial cell state in multiple cancer types, whereas its expression is strongly decreased in mesenchymal-like tumor cells. This may be explained by direct repression of *RBM47* via the EMT-inducers STAT3 and SNAIL, which are generally activated in mesenchymal-like, invasive cancer cells. While this manuscript was in preparation Sakurai *et al*. showed that expression of RBM47 is also repressed by TGF-β via direct binding of Smad3 to the *RBM47* promoter^31^. Interestingly, CRC cell lines with an intact TGF-β receptor, such as HCT15, undergo EMT after exposure to TGF-β^32^, whereas normal epithelial cells display a growth arrest. In this context it is noteworthy that the tumor microenvironment plays an important role in tumor-associated EMT, invasion, and metastasis. For example, IL-6 and TGF-β are produced and secreted by stromal cells of the tumor microenvironment^28^. Since both cytokines lead to repression of *RBM47* in tumor cells, this might represent an important mechanism as to how the tumor microenvironment promotes cancer progression.

Interestingly, comparison of the CRC cell line pair SW480/SW620, which both originate from the same patient – SW480 from the primary tumor and SW620 from a lymph node metastasis – showed an elevated expression of RBM47 in the SW620 cells. This finding might be explained by a reversion of EMT during metastasis. It has been suggested that cancer cells first switch from epithelial- to mesenchymal-like cell state (EMT) to leave the primary tumor, and then switch back to an epithelial-like state (mesenchymal to epithelial transition (MET)) to colonize distant organs and form metastases^33–35^. Accordingly, based on CCLE data and our previous findings, expression of the epithelial marker E-cadherin is higher in SW620 cells than in SW480 cells^26^. Therefore, re-expression of RBM47 after extravasation might promote metastatic colonization.

The tail-vein injection model of lung metastasis used here does not recapitulate the whole metastatic process, but rather the late stages - extravasation and colonization^36^. However, it has been shown that EMT promotes extravasation after tail vein injection of tumor cells^33^, which may explain the formation of metastases in mice injected with DLD1 CRC cells treated with RBM47-specific siRNAs. After extravasation, which is a relatively rapid process^37^, cancer cells remain in the lungs for several weeks. By this time the siRNAs are presumably too diluted in order to repress RBM47. The resulting re-expression of RBM47 might promote the formation of macro-metastases, since the epithelial state is known to promote metastatic colonization by enhancing proliferation.

Potential mechanisms of tumor suppression by RBM47 have been suggested in recent studies (see also Fig. 8f). RBM47 is a RNA binding protein that binds predominantly to introns and 3′-UTRs of its target mRNAs and regulates their stability^38^: RBM47 binds to ∼2500 mRNAs in human breast cancer cells. One of these transcripts is the *DKK1/Dickkopf1* mRNA, which encodes an inhibitor of Wnt signaling. RBM47 stabilizes *DKK1* mRNA and thereby inhibits Wnt activity. This effect could also explain how RBM47 mediates suppression of breast cancer metastases^38^. In addition, the regulation of the Wnt pathway by RBM47 was shown to play an important role during zebrafish development^39^. Since Wnt signaling has a crucial role in CRC metastasis, the Wnt-suppressing function of RBM47 via stabilization of *DKK1* may suppress CRC progression. In addition, RBM47 regulates RNA editing^40^ and splicing^41, 42^. Moreover, RBM47 was shown to suppress lung cancer growth through inhibition of Nrf2 activity^31^. RBM47 directly binds to mRNAs of Nrf2 inhibitors Cullin3 and Keap1 thereby up-regulating their expression. Given its diverse functions, the tumor suppressive properties of RBM47 may be mediated by several different targets and/or mechanisms.

Taken together, our results show that down-regulation of RBM47 is associated with and functionally important for CRC progression. Therefore, detection of RBM47 down-regulation may serve as a new prognostic marker for CRC and might represent an important diagnostic option to identify patients with poor prognosis in the future. In addition, our results suggest that restoration of RBM47 function represents a potential therapeutic strategy for treatment of metastatic CRC.

## Materials and Methods

### Cell lines, cell culture and reagents

Colorectal cancer (SW480, SW620, Caco-2), breast cancer (MCF7, MDA-MB-231), and prostate cancer (LNCaP, PC-3) cell lines were maintained in Dulbecco’s Modified Eagles Medium (DMEM, Invitrogen) containing 10% fetal bovine serum (FBS, Invitrogen). The colorectal cancer cell lines HCT-15, HT29, LS174T, HCT116 and DLD-1 were maintained in McCoy’s 5 A Medium (Invitrogen) containing 10% FBS. All cells were cultivated in presence of 100 units/ml penicillin and 0.1 mg/ml streptomycin. SiRNAs (Ambion silencer siRNA: negative control (ID#4611) and RBM47 (ID#s29090) were transfected at a final concentration of 10 nM using HiPerfect transfection reagent (Qiagen). IL-6 (Immunotools) was dissolved in water and used at a final concentration of 20 ng/ml.

### Conditional expression of *SNAIL* and *SLUG* alleles in cell pools

Stable DLD1/pRTR-SNAIL cell pools were described previously^32^. The *SLUG* cDNA was amplified by PCR from pcDNA-SLUG (a kind gift from Kou-Juey Wu, Institute of Biochemistry & Molecular Biology, National Yang-Ming University, Taiwan), verified by sequencing and cloned into the pRTR vector. Stable DLD1/pRTR cell pools were obtained transfection of DLD-1 cells with pRTR plasmids using FuGene reagent (Roche). After 24 hours, cells were transferred into media containing 4 µg/ml Puromycin for one week. Homogeneity of the derived cell pools was tested by addition of 100 ng/ml DOX for 48 hours and evaluation of GFP expression by fluorescence microscopy.

### RNA isolation and quantitative real-time PCR (qPCR)

Total RNA was isolated using the Total RNA Isolation Kit (Roche) according to manufacturer’s instructions. cDNA was generated from 1 µg total RNA per sample using the Verso cDNA synthesis kit (Thermo scientific). Quantitative real-time PCR (qPCR) was performed by using the LightCycler 480 (Roche) and the Fast SYBR Green Master Mix (Applied Biosystems). Expression was normalized using detection of *GAPDH* using the ΔΔCt method^43^. Results are represented as fold induction of the treated/transfected condition compared with the control condition Experiments were performed in triplicates. The sequences of oligonucleotides used as qPCR primers are listed in Supplemental Table S4.

### Chromatin immunoprecipitation (ChIP) assay

Cross-linking of cells was performed with 1% formaldehyde (Merck) and terminated after 5 minutes by addition of glycine at a final concentration of 0.125 M. Cells were harvested with SDS buffer (50 mM Tris pH 8.1, 0.5% SDS, 100 mM NaCl, 5 mM EDTA) and after pelleting resuspended in IP buffer (2 parts of SDS buffer and 1 part Triton dilution buffer (100 mM Tris-HCl pH 8.6, 100 mM NaCl, 5 mM EDTA, pH 8.0, 0.2% NaN_3_, 5.0% Triton X-100). Chromatin was sheered by 8 sonication cycles (HTU SONI 130, G. Heinemann) to generate DNA fragments with an average size of 700 bp for qChIP. Preclearing and incubation with polyclonal STAT3 antibody (sc-482, Santa Cruz), SNAIL antibody (#AF3639, R&D systems), or IgG control (#R-5506, Sigma or #AB-108-C, R&D systems) for 16 hours was performed as previously described^44^. Washing and reversal of cross-linking was performed as described^45^. ChIP-DNA was analyzed by qPCR and the enrichment was expressed as % input. Experiments were performed in triplicates. The sequences of oligonucleotides used as qChIP primers are listed in Supplemental Table S5.

### Western blot analysis and antibodies

Cell-lysates were collected in RIPA lysis buffer (50 mM Tris/HCl, pH 8.0, 250 mM NaCl, 1% NP40, 0.5% (w/v) sodium deoxycholate, 0.1% sodium dodecylsulfate, complete mini protease and phosphatase inhibitors (Roche). Lysates were sonicated and centrifuged for 15 min at 4 °C. Per lane 30–60 µg of whole cell lysate was separated on 10% SDS-acrylamide gels and transferred on Immobilon PVDF membranes (Millipore). For immunodetection membranes were incubated with antibodies listed in Supplemental Table S6. Signals from horse-radish-peroxidase (HRP) - coupled secondary antibodies were generated by enhanced chemiluminescence (Millipore) and recorded with a CCD camera (440CF imaging system, Eastman Kodak Co.). Intensities of protein expression signals were quantified using densitometric analysis with the Kodak Molecular Imaging Software v5.0.1.27.

### Boyden-chamber invasion assay

To analyse invasion, cell inserts (8.0 µm pore size membrane; Corning) were first coated with Matrigel (BD Bioscience) at a dilution of 3.3 ng/ml in medium without serum. Subsequently, 5 × 10^4^ cells, previously deprived of serum (0.1%) for 24 hours, were seeded on the Matrigel in the upper chamber in serum free medium. As chemo-attractant 20% FBS was placed in the lower chamber. After 48 hours, non-motile cells at the top of the filter were removed and the cells in the bottom chamber were fixed with methanol and stained with DAPI and counted using immunofluorescence microscopy. Results represent the average number of cells in five fields per membrane in triplicate inserts. Experiments were performed in triplicates.

### Wound healing assay

Cells were cultured until they reached complete confluence. Mitomycin C [10 ng/ml] was added two hours before generating a scratch using a pipette tip. After washing twice with HBSS to remove Mitomycin C and detached cells, medium was added. Images were captured on an Axiovert Observer Z.1 microscope connected to an AxioCam MRm camera using the Axiovision software (Zeiss) at the respective time-points. Experiments were performed in triplicates.

### Metastases formation in a xenograft mouse model

DLD-1 cells stably expressing Luc2 were generated as described previously^11^. 4 × 10^6^/0.2 ml Luciferase tagged cells were injected into the lateral tail vein of NOD/SCID mice using 25-gauge needles. In weekly intervals anesthetized mice were injected intraperitoneal with D-luciferin (150 mg/kg) and imaged 10 minutes after injection using the IVIS Illumina System (Caliper Life Sciences). The acquisition time was 2 minutes. 8 weeks after tail vein injection, mice were sacrificed and examined for lung metastases. For H&E stainings, lungs were fixed with 4% paraformaldehyde and 3 μm sections were stained with haematoxylin and eosin. All studies involving mice were conducted with approval by the local Animal Experimentation Committee (Regierung of Oberbayern). All experiments were performed in accordance with relevant guidelines and regulations.

### Clinical samples and immunohistochemistry

RBM47 expression was evaluated using formalin-fixed, paraffin-embedded (FFPE) colon cancer samples of 86 patients who underwent surgical tumor resection at the Ludwig-Maximilians University of Munich (LMU) between 1994 and 2005. Follow-up data were recorded by the tumor registry Munich. All tumors were located on the right side of the colon. Half of the patients had colon cancers with synchronous liver metastases, where metastasis was diagnosed by clinical imaging or liver biopsy. Controls consisted of colon cancer patients without distant metastases at the time of diagnosis and with a disease-free survival of at least 5 years after primary surgical resection. The samples of cases and controls were matched by tumor grade (according to WHO 2000), T-classification (according to TNM Classification of Malignant Tumors 2009), and tumor localization, resulting in 43 matched pairs. Tissue microarrays (TMAs) were generated with 6 representative 1 mm cores of each case. 5 µm TMA sections were prepared, deparaffinized, and stained with anti-RBM47 (Abcam ab167164) rabbit monoclonal antibody on a Ventana Benchmark XT Autostainer with UltraView Universal DAB and alkaline phosphatase detection kits (Ventana Medical Systems). RBM47 expression was scored semi-quantitatively, ranging from complete absence (score 0), weak (score 1), moderate (score 2), or strong expression (score 3). The difference in RBM47 expression between patients without distant metastases was analyzed using the Chi-square test. The study was performed with permission of the ethics committee of the Medical Faculty of the LMU. All analyses were performed in accordance with relevant guidelines and regulations.

### Analysis of expression data from public databases

TCGA expression and clinical data was obtained from the TCGA data portal (tcga-data.nci.nih.gov/tcga/tcgaDownload.jsp)^19^ and the UCSC cancer browser (https://genome-cancer.ucsc.edu)^46^. The RNA-Seq by Expectation-Maximization (RSEM) normalized expression values from the Illumina RNASeqV2 (genes) datasets were used. Expression data of cancer cell lines was obtained from The Cancer Cell line Encyclopedia (http://www.broadinstitute.org/ccle/home)^20^. The quantile normalized robust multi-array average (RMA) values assessed by the Affymetrix U133 PLUS 2.0 array were used. The CMS classification of TCGA and GSE39582 samples was obtained from Synapse (www.synapse.org), #syn2623706. Expression and clinical data of GSE39582, GSE37892, GSE24551, GSE17536, and GSE33113 datasets was downloaded from NCBI GEO (www.ncbi.nlm.nih.gov/geo).

### Statistical analysis

For CCLE analyses, cell lines from colorectal, breast, lung, bladder, and pancreatic cancer were first grouped according to their epithelial/mesenchymal characteristics: epithelial-like cell lines (expression of *CDH1* more than 10-fold higher than expression of *VIM*) and mesenchymal-like cell lines (expression of *VIM* more than 10-fold higher than expression of *CDH1*). Next, the difference in expression of all mRNAs between epithelial-like and mesenchymal-like cancer cell lines was analyzed by a Student’s t-test (p < 0.05 was considered significant). For analyses of TCGA data, the expression of each mRNA was correlated with the expression of *CDH1* and *VIM* in seven cancer entities using the Spearman non-parametric correlation coefficient. Epithelial state-associated mRNAs were defined as mRNAs that positively correlate with expression of the epithelial marker *CDH1* (r > 0.1) and negatively correlate with expression of the mesenchymal marker *VIM* (r < −0.1). Mesenchymal state-associated mRNAs were defined as mRNAs that negatively correlate with expression of the epithelial marker *CDH1* (r < −0.1) and positively correlate with expression of the mesenchymal marker *VIM* (r > 0.1). Data was analyzed and results visualized using the Multiple Experiment Viewer package^47^. Venn diagrams were designed using Venn Diagram Plotter software (https://omics.pnl.gov/software/venn-diagram-plotter). Expression of mRNA signatures was calculated as average of z-score normalized expression of all mRNAs from the signature. The statistics for Kaplan-Meier survival curves was calculated by log-rank test. For binary classification of cases (high/low expression), receiver operated characteristics (ROC) curve analysis was used to determine optimal cut-off values using the Cut-off Finder tool (http://molpath.charite.de/cutoff/assign.jsp). The classification of TCGA COAD and GSE39582 samples into epithelial- or mesenchymal-associated groups in Fig. 2a and b was performed using k-means clustering (Pearson correlation, number of clusters: 2, maximum iterations: 50). The association of *RBM47* expression with nodal status or metastasis in TCGA data was calculated by Student’s t-test. The association of RBM47 protein expression with metastasis was calculated using the chi-square test. qPCR and invasion data is expressed as mean ± SD. Differences were analyzed by a two tailed Student’s t-test. Differences in total photon flux in xenograft experiments were determined by a paired two tailed Student’s t-test. Calculations were performed using Prism5 (Graph Pad Software Inc.) and p-values ≤ 0.05 were considered as significant. * ≤ 0.05; ** ≤ 0.01; *** ≤ 0.001.

## Electronic supplementary material

Supplemental Information

Supplemental data 1

Supplemental data 2
