# Supplementary material for: Pan-cancer EMT-signature identifies RBM47 down-regulation during colorectal cancer progression
Source: Sci Rep. 2017 Jul 5;7:4687. doi: 10.1038/s41598-017-04234-2 (PMC5498532; doi:10.1038/s41598-017-04234-2)
Supplement: Supplementary file 1 — Supplemental Information [file 41598_2017_4234_MOESM1_ESM.pdf]

SUPPLEMENTAL MATERIAL

Pan-cancer EMT-signature identifies RBM47 down-regulation during colorectal cancer progression

Matjaz Rokavec, Markus Kaller, David Horst, and Heiko Hermeking

Inventory of Supplemental Information

- **Supplemental Figure S1.** Distribution of Vimentin (VIM) and E-cadherin (CDH1) expression in CCLE colorectal cancer cell lines and TCGA colon cancer primary tumors.

- **Supplemental Figure S2.** Venn diagrams showing overlaps between identified CCLE and TCGA epithelial or mesenchymal state-associated mRNAs**.**

- **Supplemental Figure S3.** Association of *RBM47* expression with overall and relapse free survival in the TCGA pan-cancer dataset

- **Supplemental Table S1.** Comparison of the shared TCGA/CCLE epithelial state-associated mRNA signature with published epithelial-signatures.

- **Supplemental Table S2.** Association of RBM47 expression with clinical and pathological variables

- **Supplemental Table S3.** RBM47 protein expression in tumors and adjacent normal mucosa

- **Supplemental Table S4**. Oligonucleotides used for qPCR

- **Supplemental Table S5**. Oligonucleotides used for qChIP

- **Supplemental Table S6**. List of antibodies

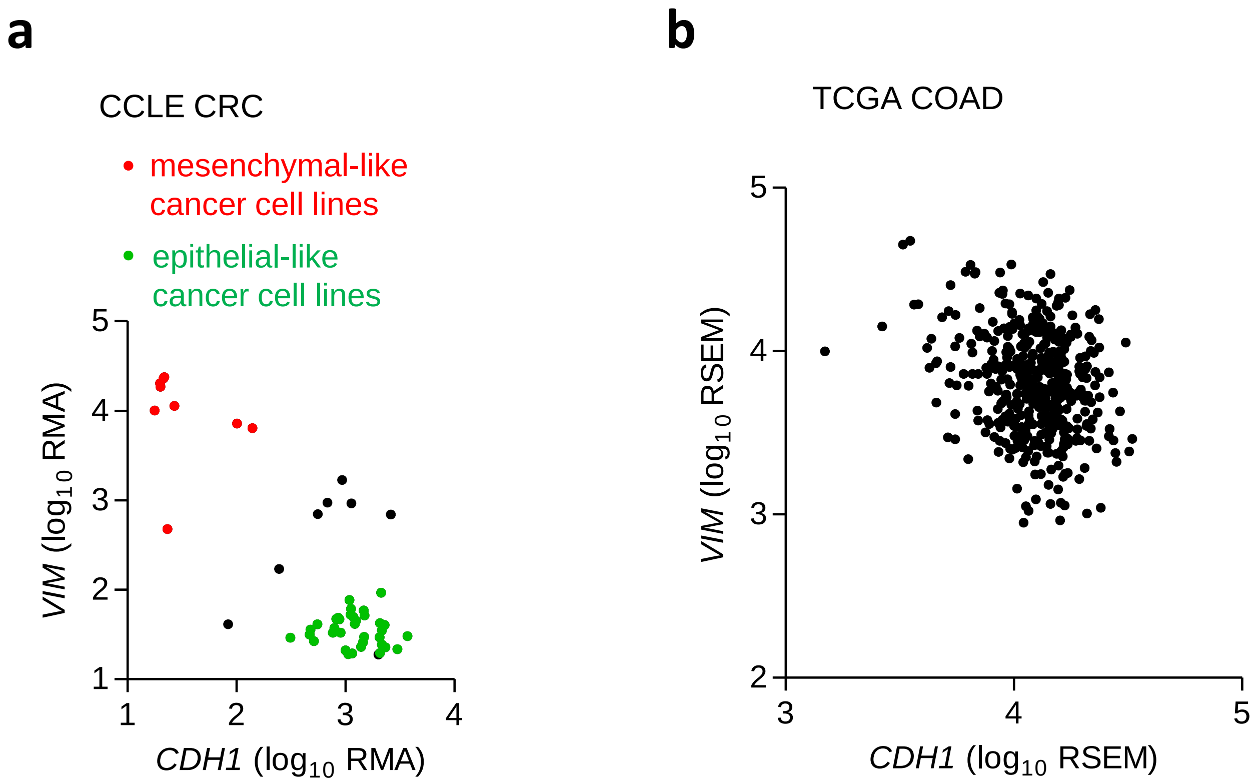

Supplemental Figure S1. Distribution of Vimentin (*VIM*) and E-cadherin (*CDH1*) mRNA expression in CCLE colorectal cancer cell lines (**a**) and TCGA colon cancer primary tumors (**b**).

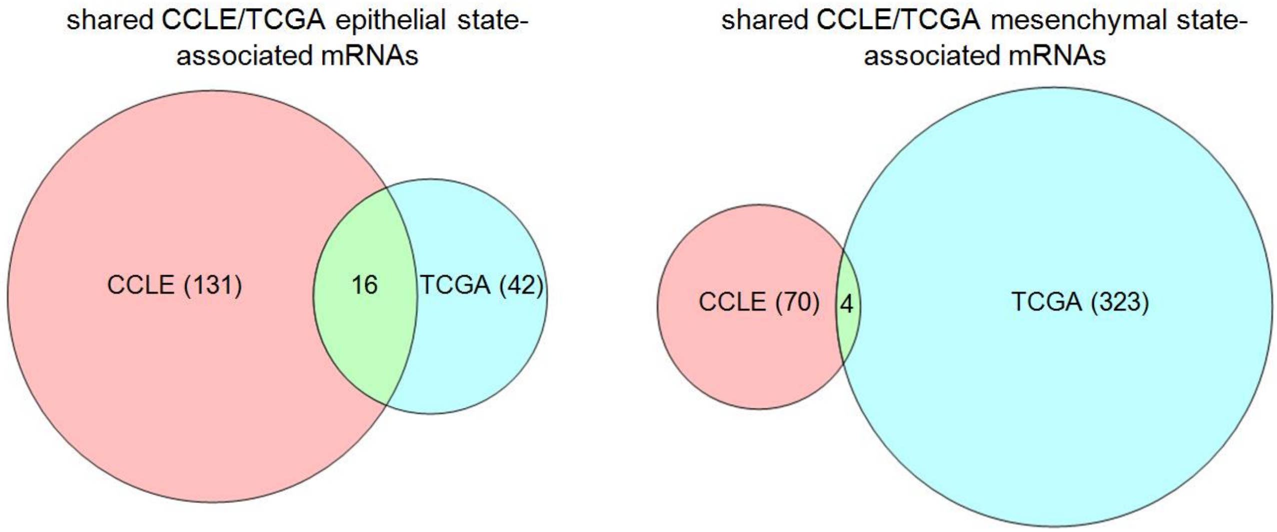

Supplemental Figure S2. Venn diagrams showing overlaps between identified CCLE and TCGA epithelial or mesenchymal state-associated mRNAs**.**

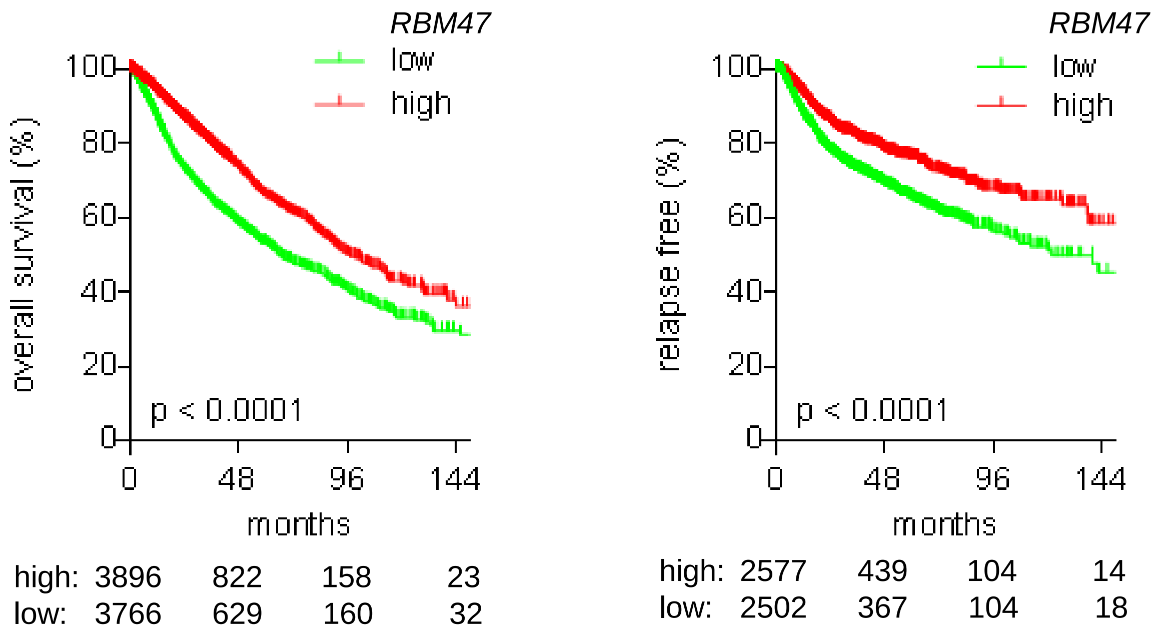

Supplemental Figure S3. Association of *RBM47* expression with overall and relapse free survival in the TCGA pan-cancer dataset.

Supplemental Table S1. Comparison of the shared TCGA/CCLE EMT-associated mRNA signature with published EMT-signatures.

mRNA	Tan, 2014	Taube, 2010	Groger, 2012	Mak, 2016
epithelial state-associated mRNAs				
AFTPH				
CDH1	*	*	*	*
CDS1	*	*	*	*
EPCAM	*		*	
ESRP1	*			*
ESRP2	*			*
FA2H				
HOOK1		*		*
LNX1				
MAP7	*		*	*
MAPK13	*			
MARVELD2				*
MARVELD3				*
MYO5B				*
MYO5C	*	*		
RBM47	*			
mesenchymal state-associated mRNAs				
LGALS1				
P4HA3				
UROD				
VIM	*	*	*	*

*, mRNA is present in the indicated EMT-associated signature

Tan, T. Z. *et al.* Epithelial-mesenchymal transition spectrum quantification and its efficacy in deciphering survival and drug responses of cancer patients. *EMBO Mol Med* **6**, 1279-1293 (2014).

Taube, J. H. *et al.* Core epithelial-to-mesenchymal transition interactome gene-expression signature is associated with claudin-low and metaplastic breast cancer subtypes. *Proc Natl Acad Sci U S A* **107**, 15449-15454 (2010).

Groger, C. J., Grubinger, M., Waldhor, T., Vierlinger, K. & Mikulits, W. Meta-analysis of gene expression signatures defining the epithelial to mesenchymal transition during cancer progression. *PLoS One* **7**, e51136 (2012).

Mak, M. P. *et al.* A Patient-Derived, Pan-Cancer EMT Signature Identifies Global Molecular Alterations and Immune Target Enrichment Following Epithelial-to-Mesenchymal Transition. *Clin Cancer Res* **22**, 609-620 (2016).

Supplemental Table S2. Association of RBM47 expression with clinical and pathological variables in the case control cohort of primary CRCs with and without liver metastasis

Characteristics	Total	RBM47		p-value
		Low (scores 0, 1)	High (scores 2, 3)	
All patients	86	55 (64.0)	31 (36.0)	
Age (y, Median 68)				
< 68	45	29 (64.4)	16 (35.6)	0.92
≥ 68	41	26 (63.4)	15 (36.6)	
Sex				
Male	41	27 (65.9)	14 (34.1)	0.73
Female	45	28 (62.2	17 (37.8)	
Tumor size (UICC)				
T2	8	4 (50.0)	4 (50.0)	0.68
T3	63	41 (65.1)	22 (34.9)	
T4	15	10 (66.7)	5 (33.3)	
Nodal status				
N0	38	19 (50.0)	19 (50.0)	**0.016**
N+	48	36 (75.0)	12 (25.0)	
Metastasis (Liver)				
M0	43	21 (48.8)	22 (51.2)	**0.0035**
M1	43	34 (79.1)	9 (20.9)	
Tumor grade (WHO)				
Low	28	17 (60.7)	11 (39.3)	0.66
High	58	38 (65.5)	29 (34.5)	

Percent values are given in parentheses

Supplemental Table S3. RBM47 protein expression in tumors and adjacent normal mucosa.

	RBM47 protein expression		
Criteria	Normal mucosa > Tumor	Normal Mucosa = Tumor	Normal Mucosa < Tumor
Patient samples*	16	9	6

* Number of patients following the indicated criteria of RMB47 expression in tumors and adjacent normal mucosa

Supplemental Table S4. **Oligonucleotides used for qPCR**

gene	forward (5' – 3')	reverse (5' – 3')
GAPDH	TGTTGCCATCAATGACCCCTT	CTCCACGACGTACTCAGCG
RBM47	ATCAGCAATCCTTGGCTCAC	CCTTGGGATTCCTCTGTTCA
CDH1	CCCGGGACAACGTTTATTAC	GCTGGCTCAAGTCAAAGTCC
OCLN	TTTGTGGGACAAGGAACACA	TATGCCATGGGACTGTCAAC
CLDN1	GGCAGATCCAGTGCAAAGTC	GGTGGCCACAAAGATTGCTA
CLDN3	GGGACTTCTACAACCCCGTG	CCGTGTACTTCTTCTCGCGT
ZO-1	CAGGAAATCTATTTCAAGGTCTGC	CATCACCAAAGGACTCAGCA
SNAIL	GCACATCCGAAGCCACAC	GGAGAAGGTCCGAGCACAC

Supplemental Table S5. Oligonucleotides used for qChIP

gene	forward (5' – 3')	reverse (5' – 3')
STAT3 BDS1	GGTTCCCTCCCTCTCTCCT	CGAGAGACAACCTTGCTACTG
STAT3 BDS2	GGCAACTTCCCGTAAATCTG	AATGTGGCACTGGCCTTTAG
SNAIL BDS-1	GCCCCAAAAGTCACATGC	CGTCTGACTCGGACAGATGC
AchR	CCTTCATTGGGATCACCACG	AGGAGATGAGTACCAGCAGGTTG

Supplemental Table S6. List of antibodies

Primary antibodies

epitope	catalog no.	company	use	dilution	source
RBM47	# ab167164	Abcam	WB	1:3000	rabbit
E-cadherin	#334000	Invitrogen	WB	1:1000	mouse
α-tubulin	#T-9026	Sigma	WB	1:1000	mouse
SLUG	#sc-15391	Santa Cruz	WB	1:1000	rabbit
SNAIL	#3879S	Cell Signaling	WB	1:1000	rabbit
SNAIL	#AF3639	R&D Systems	ChIP		goat
STAT3	#sc-482	Santa Cruz	WB<br>ChIP	1:1000	rabbit
p-STAT3 Tyr705	#9145 XP	Cell Signaling	WB	1:1000	rabbit
Rabbit IgG	#R-5506	Sigma	ChIP		rabbit
Goat IgG	#AB-108-C	R&D Systems	ChIP		goat

Secondary antibodies or conjugates

name	catalog no.	company	use	dilution	source
anti-mouse HRP	# W4021	Promega	WB	1:10.000	goat
anti-rabbit HRP	# A0545	Sigma	WB	1:10.000	goat
